# Effect of Corrosion Inhibitor on Properties and Microstructure of Self-Compacting Concrete

**DOI:** 10.3390/ma19153198

**Published:** 2026-07-27

**Authors:** Yuedong Wu, Haojie Li, Changsheng Yue, Ying Zhang, Lei Zhang, Wen Lv, Yining Kang, Shuo Zhang, Tianlei Wang

**Affiliations:** 1 State Key Laboratory of Iron and Steel Industry Environmental Protection, Central Research Institute of Building and Construction Co., Ltd., Beijing 102629, China; wuyuedong2015@163.com (Y.W.); yuecs163@163.com (C.Y.); lvwen10@126.com (W.L.); kangyining2020@163.com (Y.K.); 13146560128@163.com (S.Z.); 2 Tianjin Key Laboratory of Building Green Functional Materials, School of Materials Science and Engineering, Tianjin Chengjian University, Tianjin 300384, China; 15003879405@163.com (H.L.);

**Keywords:** self-compacting concrete, corrosion inhibitor, mechanical properties, electric flux, pore structure

## Abstract

The premature deterioration of reinforced concrete structures caused by steel reinforcement corrosion remains a major challenge to long-term structural durability. This study systematically investigates the effects of corrosion inhibitor dosage on the fresh properties, mechanical performance, chloride ion penetration resistance, and capillary water absorption of self-compacting concrete (SCC). The evolution of the pore structure is characterized using low-field nuclear magnetic resonance (LF-NMR) and X-ray computed tomography (X-CT), and the proportions of pores within different equivalent spherical diameter ranges are quantified. In addition, the microstructural characteristics are examined by scanning electron microscopy (SEM). The results show that the incorporation of the corrosion inhibitor increases the viscosity of fresh SCC, resulting in reductions in slump. In general, the corrosion inhibitor reduces both the compressive strength and splitting tensile strength of SCC, with the smallest strength reduction observed at a corrosion inhibitor dosage of 2 wt%. All mixtures containing the corrosion inhibitor exhibit lower electric flux and water absorption than the control mixture, indicating improved resistance to chloride ion penetration and capillary water ingress. The combined LF-NMR, X-CT, and SEM results indicate that an appropriate corrosion inhibitor dosage may optimize the spatial distribution of hydration products, refine the pore structure, reduce total porosity, and suppress the formation of macropores. Overall, a dosage of 2 wt% provides the most favorable balance among workability, mechanical properties, durability, and microstructural compactness. These findings provide experimental support and technical guidance for the mixture design of durable SCC used in aggressive environments, including marine and salt-lake regions.

## 1. Introduction

Concrete is the most widely used construction material in the world. Despite its widespread application and excellent structural performance in roads, bridges, buildings, and other civil infrastructure, the durability of reinforced concrete structures remains a major concern. In particular, the premature deterioration caused by steel reinforcement corrosion is one of the most critical factors limiting its long-term service life and structural performance. Steel reinforcement corrosion is fundamentally an electrochemical process. When the passivation film on the steel surface is destroyed by chloride ion penetration or concrete carbonation, corrosion reactions are initiated. The accumulation of corrosion products causes volumetric expansion, which generates internal tensile stresses within the concrete and ultimately leads to cracking and structural deterioration [1,2]. Therefore, enhancing the corrosion resistance of steel reinforcement in concrete is of great importance for extending the service life of concrete structures.

The extent of damage to reinforced concrete structures caused by steel reinforcement corrosion depends on the corrosion environment and the corresponding corrosion mechanism [3]. Yang et al. [4] reported that higher-strength concrete exhibited lower electrochemical corrosion of embedded steel reinforcement. In addition, the corrosion rate decreased with increasing steel bar diameter. Khoshroo et al. [5] investigated the effects of pre-rusted reinforcing bars with varying corrosion levels on the performance of self-compacting concrete (SCC) and ordinary concrete. Their results showed that the denser microstructure and lower permeability of SCC provided superior resistance to the ingress of chlorides and other aggressive agents, thereby mitigating the strength deterioration associated with steel reinforcement corrosion more effectively than ordinary concrete.

Although the dense microstructure and low permeability of SCC provide improved resistance to the ingress of aggressive agents compared with ordinary concrete, the corrosion of embedded steel reinforcement remains one of the primary factors limiting the long-term durability of SCC-reinforced concrete structures. Consequently, effective corrosion protection of reinforcing steel is crucial for fully exploiting the durability advantages of SCC and further improving the service life and structural performance of SCC-reinforced concrete.

Among the various corrosion protection strategies, including cathodic protection, concrete surface coatings, and admixed corrosion inhibitors, internal corrosion inhibitors have attracted considerable attention because of their ease of application, relatively low cost, and ability to provide distributed protection for embedded steel reinforcement [6,7,8]. Conventional nitrite-based inhibitors can effectively enhance the corrosion resistance of reinforcing steel and may also improve the early-age strength of concrete. However, excessive nitrite contents may promote the formation of abundant needle-like crystalline products with brittle fracture characteristics at later ages. This may interfere with the formation and development of calcium silicate hydrate (C-S-H) gel and Ca(OH)_2_, thereby adversely affecting later-age strength, toughness, and long-term durability [9,10,11].

In recent years, environmentally friendly corrosion inhibitors derived from plant extracts have been extensively investigated. Extracts obtained from *Cichorium intybus* leaves [12], caffeic acid [13], ginger [14], hawthorn and *Corchorus olitorius* [15], and green tea [16] have demonstrated varying degrees of corrosion inhibition. Nevertheless, many plant-based inhibitors are susceptible to chemical degradation or compositional instability in the highly alkaline concrete environment. Consequently, their long-term stability and corrosion inhibition efficiency are often lower than those of conventional nitrite-based inhibitors. Inorganic inhibitors based on phosphate and molybdates have also received considerable attention. Phosphate-based inhibitors, such as cerium tris(2-ethylhexyl) phosphate particles [17] and Na_3_PO_4_ [18,19], as well as molybdate-based inhibitors [20], can enhance the corrosion resistance of reinforcing steel by modifying the chemical environment of the pore solution and promoting the formation of adsorption, passivation, or precipitation films under chloride, sulfate, and carbonation exposure. However, the effectiveness of these inhibitors depends strongly on the formation, continuity, and long-term stability of the protective films on the steel surface.

Organic corrosion inhibitors have subsequently been developed to provide more effective and durable protection through molecular adsorption. Cai et al. [21] synthesized three imidazolium ionic liquids containing polar functional groups at the N1 position and alkyl chains at the N3 position and evaluated their corrosion inhibition performance for carbon steel in a simulated concrete pore solution consisting of saturated Ca(OH)_2_ and 0.3 mol/L NaCl. The ionic liquids were found to adsorb onto the steel surface through both physical and chemical interactions, thereby suppressing corrosion product formation and producing a protective adsorption film. This mixed-type inhibition behavior provides basis for the ionic liquids’ potential application in electrochemical injection-based corrosion protection.

Tian et al. [22] investigated the effects of organic corrosion inhibitors on the corrosion behavior of reinforcing steel subjected to the combined action of freeze–thaw cycling and chloride attack in marine environments. Alcohol-amine-, carboxylic-acid-, and ester-based inhibitors improved the salt-freezing resistance of concrete. In particular, alcohol-amine inhibitors enhanced the stability of the passivation film on the steel surface, whereas carboxylic-acid inhibitors competed with or displaced adsorbed chloride ions. Both mechanisms effectively delayed the depassivation of reinforcing steel. Liu et al. [23] developed a composite organic inhibitor by combining triethanolamine and triisopropanolamine at a mass ratio of 4:6. The resulting inhibitor simultaneously suppressed cathodic and anodic reactions and formed a protective film through combined physical and chemical adsorption, thereby effectively reducing steel corrosion in concrete.

Hang et al. [24] evaluated triethanolamine and water-soluble imidazolidine inhibitors for carbon steel exposed to simulated concrete pore solutions containing mixed salts. Both inhibitors effectively suppressed the rapid corrosion of carbon steel. Their protective action was attributed primarily to the adsorption of organic molecules on the steel surface, which formed a barrier against the transport and accumulation of aggressive ions. He et al. [25] reported that a composite amino-alcohol inhibitor achieved a corrosion inhibition efficiency of 95.8% in a simulated concrete pore solution, exceeding the 93.2% efficiency obtained using conventional calcium nitrite. The amino-alcohol inhibitor predominantly suppressed the cathodic reaction and retained considerable inhibition efficiency even after destabilization of the original passivation film. Further tests indicated that the inhibitor improved compactness, resistance to fluid penetration, and the degree of cement hydration, thereby restricting the ingress of chloride ions and water and delaying reinforcement corrosion.

Liu et al. [26] studied a carboxylate inhibitor composed of benzoic acid and dimethylethanolamine in a simulated concrete pore solution containing 0.6 mol/L chloride ions. The inhibitor increased the critical chloride threshold for carbon-steel depassivation, reduced the actively corroding area, and remained effective after 120 days of immersion. Its long-term protective mechanism involved rapid molecular adsorption and interfacial buffering during the initial stage, followed by the gradual formation of a deposited protective layer during prolonged exposure. The protection mechanism enabled the inhibitor to reduce the corrosion rate even under long-term exposure to chloride-enriched pore solutions.

More generally, amino-alcohol-based inhibitors protect reinforcing steel through the physical and chemical adsorption of organic molecules and may additionally regulate cement hydration, refine the concrete pore structure, and reduce transport pathways for aggressive species [27,28,29]. The electromigration behavior of ethanolamine in carbonated concrete has also been investigated [30]. Following electromigration treatment, the electrochemical response of the reinforcing steel changed markedly, and both corrosion activity and corrosion rate decreased. Variations in the pore-solution chemistry and ionic concentrations indicated that ethanolamine had migrated from the concrete surface to the reinforcement and accumulated at the steel–concrete interface, where it promoted the formation or restoration of a protective film.

Despite the promising performance of individual organic and inorganic inhibitors, single-component systems may provide insufficient protection under complex exposure conditions. Composite inhibitors combining amino alcohols with phosphates can exploit complementary adsorption, passivation, and pore-solution-modification mechanisms, thereby enhancing the stability of the protective film on reinforcing steel and improving corrosion resistance [31,32,33].

However, existing studies have mainly focused on the electrochemical behavior of reinforcing steel, whereas the effects of corrosion inhibitors on the fresh properties, mechanical performance, durability, and microstructure of concrete have received comparatively limited attention, particularly for SCC. Moreover, corrosion inhibitors may alter cement hydration and pore-structure evolution, but the relationships among inhibitor dosage, microstructural development, and macroscopic performance remain insufficiently understood. To address these gaps, this study systematically investigates the effects of different dosages of an amino alcohol-phosphate composite corrosion inhibitor on the workability, mechanical properties, durability, and microstructure of SCC. The objectives are to determine an appropriate inhibitor dosage, clarify its influence on cement hydration and pore-structure evolution, and establish the relationship between microstructural characteristics and macroscopic performance. The findings are expected to provide a theoretical basis and technical guidance for the mixture design of high-durability SCC.

## 2. Experimental

### 2.1. Materials

P.O. 42.5 ordinary Portland cement produced by China Shanshui Cement Group Limited (Jinan, China) was used. Its chemical composition and physical and mechanical properties comply with GB 175-2023 [34]. Fly ash (Class F, grade I; Huadian Laizhou Power Station, Yantai, China) and ground granulated blast-furnace slag (S95 grade; Qingdao Runyi Fengtai New Material Technology Co., Ltd., Qingdao, China) were used as mineral admixtures. Natural medium sand with Zone II grading was used as the fine aggregate, with a fineness modulus between 2.6 and 2.9 and an apparent density of 2510 kg/m^3^. The coarse aggregate was 5–15 mm continuously graded basalt gravel with an apparent density of 3010 kg/m^3^. A polycarboxylate-based high-performance water reducer (Sobute New Materials Co., Ltd., Nanjing, China) was used, with a solid content of 20% and a water reduction rate of approximately 28%. The corrosion inhibitor used in this study was supplied by Sobute New Materials Co., Ltd. It is a composite inhibitor containing 17% active ingredients, primarily triethanolamine, triisopropanolamine, and a small amount of sodium phosphate. It has a density of 1.05 g/cm^3^, a viscosity of 15 mPa·s at 25 °C, and a pH value of 9.6.

### 2.2. Mix Proportions

To systematically investigate the influence of the corrosion inhibitor on the performance of SCC, the corrosion inhibitor was incorporated by replacing an equivalent mass of cementitious materials. The mix proportions are listed in Table 1. ZX-0 is the control mixture without a corrosion inhibitor. In mixtures ZX-1 to ZX-4, the corrosion inhibitor dosages were 1 wt%, 2 wt%, 3 wt%, and 4 wt%, respectively, based on the total mass of cementitious materials.

### 2.3. Characterization

The workability of fresh SCC was evaluated in accordance with JGJ/T 283-2012 [35]. The passing ability of SCC was assessed based on the slump flow and J-ring flow diameters. The J-ring step height was not measured and is therefore recognized as a limitation of the present study.

To evaluate the mechanical properties, cubic compressive strength (100 mm × 100 mm × 100 mm) and splitting tensile strength (150 mm × 150 mm × 150 mm) were measured after 3, 7, and 28 days of standard curing in accordance with GB/T 50081-2019 [36].

The 28-day water absorption test was also carried out with reference to GB/T 50081-2019. In addition, in accordance with GB/T 50082-2024 [37], the electric flux of the cylindrical specimens (100 mm in diameter and 50 mm in height) cured for 28 days was measured over a 6 h period to evaluate the resistance of concrete to chloride ion penetration.

The pore characteristics of the SCC specimens were characterized using LF-NMR and X-CT. LF-NMR measurements were performed using an LMR-30 analyzer (Beijing Larmor Technology Co., Ltd., Beijing, China), and the transverse relaxation time (T_2_) distributions of the saturated specimens were determined. The measurements were conducted at a resonance frequency of 7.1585 MHz, with a sampling bandwidth of 0.2 MHz, 1024 sampling points, and eight repeated scans. X-CT analysis was carried out using a high-performance X-ray imaging system (nanoVoxel-2000, Sanying Precision Instruments Co., Ltd., Tianjin, China). The specimens were scanned at an X-ray tube voltage of 150 kV and a power of 25 W. The acquired projection images were reconstructed using the instrument’s built-in software VoxelStudio Recon (Version 2.5.1.25), and porestructure parameters, including pore-size distribution and total porosity, were subsequently quantified using the image processing and analysis software Dragonfly 3D World (Version 2024.1).

Except for porosity, the standard deviation (SD) was calculated as SD = $\surd [\Sigma (x_{i}-\bar{x})²/(n-1)]$, where $x_{i}$ is an individual measured value, $\bar{x}$ is the arithmetic mean, and n is the number of replicate specimens. Three replicate specimens (*n* = 3) were tested for each group, and the error bars in the figures represent the standard deviation. Statistical differences among the groups were evaluated using one-way analysis of variance (ANOVA), followed by Tukey’s honestly significant difference (HSD) post hoc test. Differences were considered statistically significant at *p* < 0.05.

## 3. Results and Discussion

### 3.1. Workability

**Figure 1:**
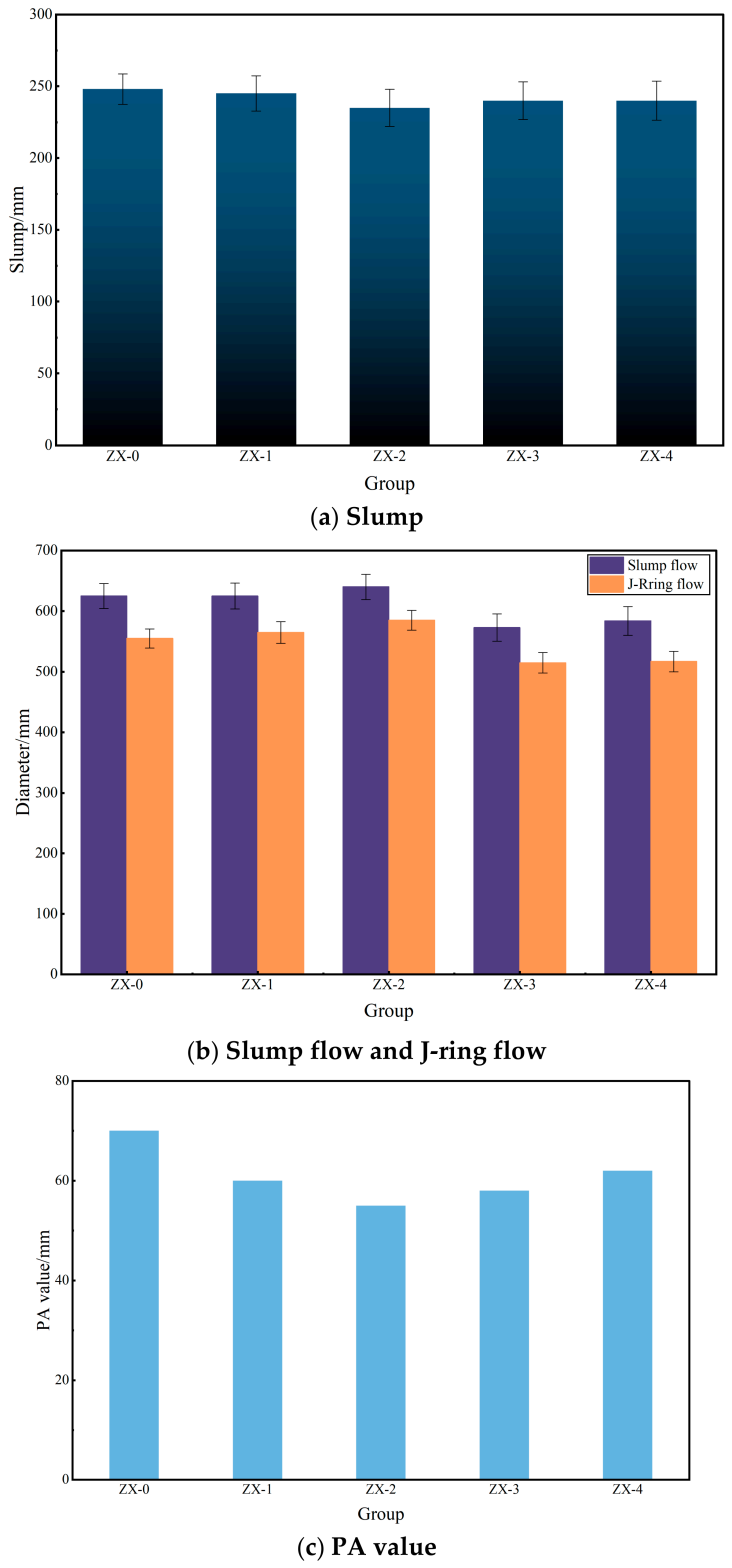
Effect of corrosion inhibitor dosage on workability of SCC.

Figure 1 illustrates the effects of corrosion inhibitor dosage on the slump, slump flow, J-ring flow, and PA value of fresh SCC, where the PA value is defined as the difference between the slump flow and J-ring flow. As shown in Figure 1a, the incorporation of the corrosion inhibitor generally reduces the slump relative to that of ZX-0. One-way analysis of variance shows that inhibitor dosage has a statistically significant effect on slump (*F*(4, 10) = 6.45, *p* = 0.007). Tukey’s HSD post hoc test further shows that the slump of ZX-2 is significantly lower than those of ZX-0 and ZX-1 (*p* < 0.05), whereas no statistically significant differences are observed among ZX-0, ZX-1, ZX-3, and ZX-4. The reduction in slump may be attributed to changes in the ionic environment and rheological behavior of the fresh cementitious suspension after inhibitor incorporation. The dissociation of amino alcohol molecules in the inhibitor raises the ionic strength of the pore solution, which may enhance interparticle flocculation among cement particles and increase the apparent viscosity of the paste, thus decreasing the flowability of the fresh mixture [26]. Nevertheless, the slump values of all inhibitor-containing mixtures remain above 235 mm, indicating that the mixtures retain satisfactory fluidity. The incorporation of the corrosion inhibitor also affects the slump flow of SCC, as shown in Figure 1b. The slump flow of ZX-1 is comparable to that of ZX-0, whereas that of ZX-2 increases to 640 mm. One-way ANOVA indicates that inhibitor dosage has a statistically significant effect on slump flow (*F*(4, 10) = 5.35, *p* = 0.013). Tukey’s HSD post hoc test further shows that the slump flow of ZX-2 is significantly higher than those of ZX-3 and ZX-4 (*p* < 0.05). This phenomenon may be associated with the surface-active behavior of the amino alcohol components in the inhibitor, which can adsorb onto cement particles, modify interfacial interactions, and promote particle dispersion. However, when the inhibitor dosage is increased to 3 wt% and 4 wt%, the slump flow decreases, possibly because the increased viscosity of the liquid phase and intensified interparticle interactions restrict the mobility of the fresh mixture.

The J-ring flow values of all mixtures exceed 510 mm, indicating satisfactory passing ability through congested reinforcement. Among all groups, ZX-2 exhibits the highest J-ring flow of 585 mm. One-way ANOVA shows that inhibitor dosage has a statistically significant effect on J-ring flow (*F*(4, 10) = 10.08, *p* = 0.0013). Tukey’s HSD post hoc test indicates that the J-ring flow of ZX-2 is significantly higher than those of ZX-3 and ZX-4 (*p* < 0.05), demonstrating that ZX-2 exhibits the most favorable filling and passing performance among the test groups.

The passing ability of SCC is further evaluated using the PA value, defined as the difference between slump flow and J-ring flow. As shown in Figure 1c, the control mixture ZX-0 exhibits the highest PA value, indicating the greatest reduction in flow when passing through the J-ring and, consequently, the highest susceptibility to blocking. In contrast, all inhibitor-containing mixtures exhibit lower PA values, suggesting that the incorporation of the corrosion inhibitor may improve the passing ability and blocking resistance of SCC. Given the limited sensitivity of the conventional slump test for SCC, the workability of the mixtures was primarily evaluated based on the slump flow and J-ring flow results.

Overall, the corrosion inhibitor has a dosage-dependent effect on the workability of SCC. A moderate dosage, particularly that used in ZX-2, improves slump flow and J-ring flow, whereas higher dosages reduce flowability, likely because of the increased viscosity and altered interparticle interactions within the fresh paste. Nevertheless, all mixtures maintain satisfactory flowability and passing ability. The lower PA values of the inhibitor-containing mixtures further indicate improved resistance to blocking compared with the control mixture.

### 3.2. Mechanical Properties

#### 3.2.1. Compressive Strength

**Figure 2:**
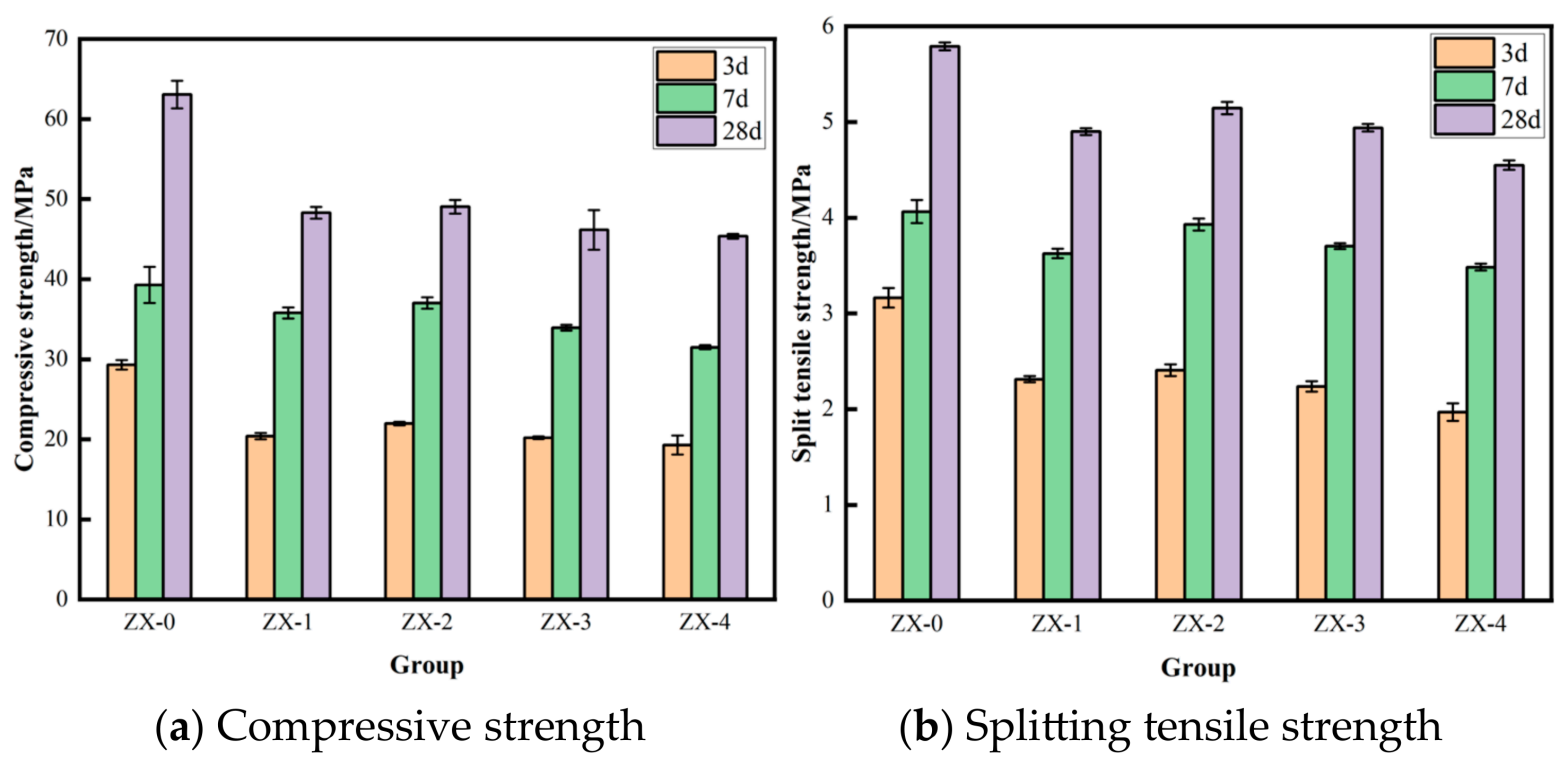
Effect of corrosion inhibitor dosage on mechanical properties of SCC.

To investigate the effect of corrosion inhibitor dosage on the mechanical properties of SCC, the compressive strengths of all mixtures were measured after 3, 7, and 28 days of curing, as shown in Figure 2a. One-way ANOVA indicates that inhibitor dosage has a statistically significant effect on compressive strength at all curing ages: 3 days, *F*(4, 10) = 124.9, *p* < 0.001; 7 days, *F*(4, 10) = 28.9, *p* < 0.001; 28 days, *F*(4, 10) = 204.7, *p* < 0.001. Tukey’s HSD post hoc test further shows that, at 28 days, the compressive strength of ZX-2 is significantly higher than those of ZX-3 and ZX-4 (*p* < 0.05), while no statistically significant difference is observed between ZX-1 and ZX-2 (*p* > 0.05).

Overall, increasing the corrosion inhibitor dosage from 0 to 4 wt% results in lower compressive strength at all curing ages, indicating an adverse effect of excessive inhibitor incorporation on the compressive strength development of SCC. This reduction may be related to the adsorption of amino alcohol molecules on cement particle surfaces. By occupying reactive sites and forming complexes with ionic species in the pore solution, the corrosion inhibitor may retard the hydration of C_3_S and C_2_S, thereby reducing the formation of early hydration products and delaying strength development [27].

The compressive strengths of ZX-2 at 3, 7, and 28 days are 22.0 MPa, 37.1 MPa, and 49.1 MPa, respectively. Among the inhibitor-containing mixtures, ZX-2 exhibits the smallest reduction in compressive strength relative to the control mixture, ZX-0, at all curing ages, while its compressive strength is not statistically different from that of ZX-1. This result suggests that an appropriate inhibitor dosage may partially alleviate the adverse effects of delayed hydration by promoting a more uniform distribution of hydration products and enabling C-S-H gel to fill the pore space more effectively. However, when the inhibitor dosage increases to 3 wt% and 4 wt%, the retardation of cement hydration becomes dominant, resulting in a further reduction in compressive strength.

#### 3.2.2. Splitting Tensile Strength

The splitting tensile strength of SCC was also measured at curing ages of 3, 7, and 28 days. As shown in Figure 2b, the effect of corrosion inhibitor dosage on splitting tensile strength is generally consistent with that observed for compressive strength. The 28-day splitting tensile strength of the control mixture, ZX-0, reaches 5.8 MPa, whereas those of the inhibitor-containing mixtures ZX-1 to ZX-4 range from 4.6 MPa to 5.2 MPa. One-way ANOVA indicates that inhibitor dosage has a statistically significant effect on splitting tensile strength at all curing ages: 3 days, *F*(4, 10) = 39.5, *p* < 0.001; 7 days, *F*(4, 10) = 20.6, *p* < 0.001; 28 days, *F*(4, 10) = 70.9, *p* < 0.001. Among the inhibitor-containing mixtures, ZX-2 exhibits the highest splitting tensile strength at each curing age. At 28 days, the splitting tensile strength of ZX-2 is 5.2 MPa, which is significantly higher than that of ZX-4 (4.6 MPa) (*p* < 0.05), according to Tukey’s HSD post hoc test.

The reduction in splitting tensile strength after inhibitor incorporation may be related to early-age hydration retardation and the resulting microstructural defects that persist during subsequent curing. The adsorption of inhibitor molecules on cement particles can reduce the formation of hydration products and weaken the internal bonding of the cementitious matrix, thereby decreasing its resistance to tensile stress [38]. Nevertheless, the comparatively high strength of ZX-2 suggests that an appropriate inhibitor dosage may partially mitigate the adverse effects of hydration retardation by promoting a relatively uniform distribution of hydration products and maintaining matrix compactness. At higher dosages, particularly in ZX-3 and ZX-4, the stronger retardation of cement hydration may lead to less compact regions and weaker interfacial bonding within the matrix, resulting in a further reduction in splitting tensile strength.

### 3.3. Electric Flux

**Figure 3:**
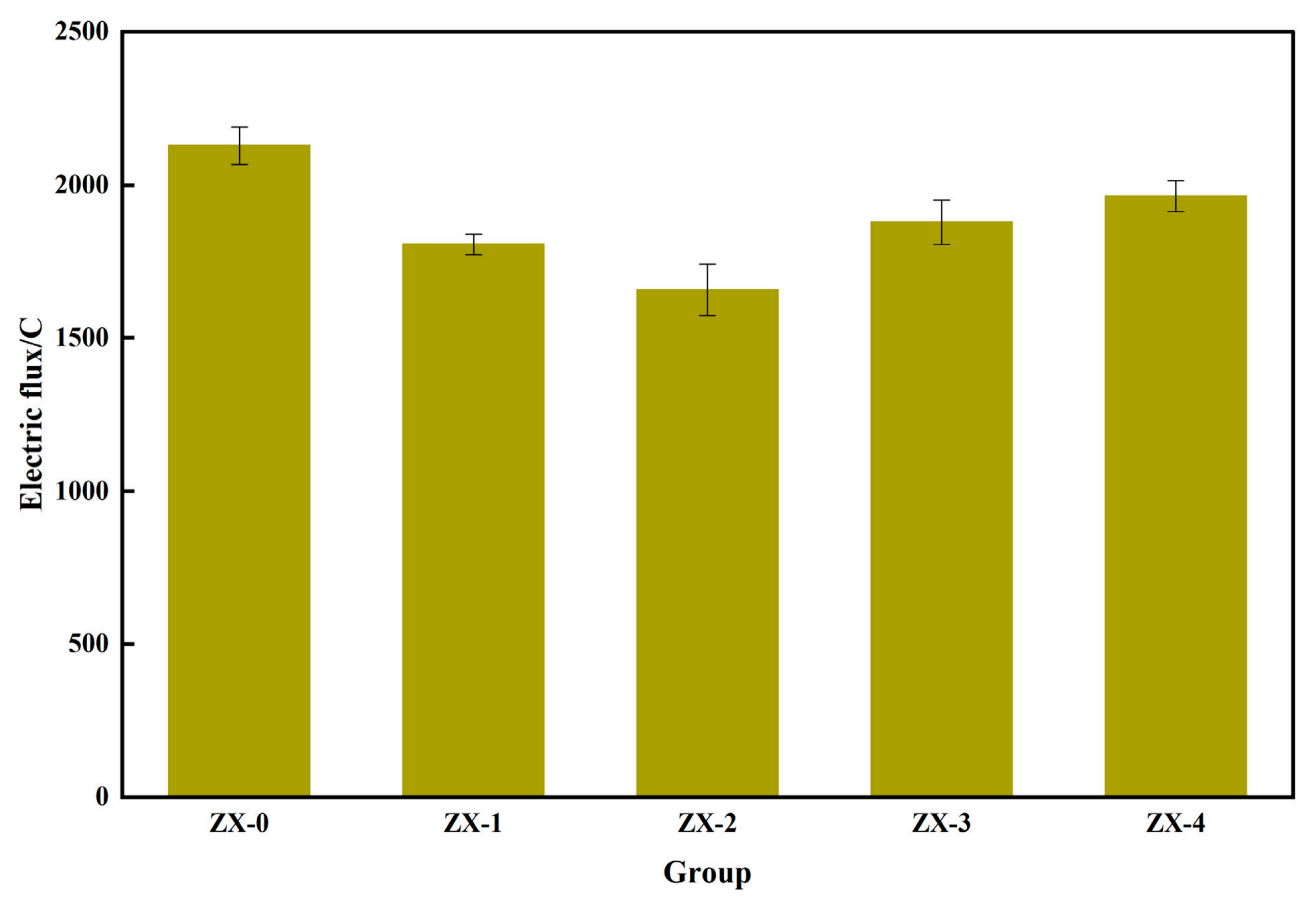
Effect of corrosion inhibitor dosage on electric flux of SCC.

The electric flux reflects the resistance of concrete to chloride ion penetration. A lower electric flux indicates greater resistance to ionic transport through the pore network and, consequently, better resistance to chloride ingress. Figure 3 shows the electric flux results of all groups. One-way ANOVA indicates that inhibitor dosage has a statistically significant effect on the electric flux (*F*(4, 10) = 27.3, *p* < 0.001). Tukey’s HSD post hoc test shows that ZX-2 exhibits the lowest electric flux of 1628 C, which is significantly lower than those of ZX-0 and ZX-4, with values of 2108 C and 1964 C, respectively (*p* < 0.05).

The incorporation of the corrosion inhibitor reduces the electric flux values to varying degrees relative to the control mixture, indicating an overall improvement in the resistance of SCC to chloride ion penetration. With increasing inhibitor dosage, the electric flux initially decreases and then increases. Specifically, the value decreases from 2108 C for ZX-0 to 1786 C for ZX-1 and reaches a minimum of 1628 C for ZX-2. This improvement may be attributed to the ability of an appropriate inhibitor dosage to promote a more uniform distribution of hydration products, refine the pore structure, and reduce pore connectivity, thereby restricting chloride transport pathways. In addition, the polar functional groups in the organic components of the corrosion inhibitor, such as hydroxyl and amino groups, may adsorb onto the pore surfaces and modify the surface-charge characteristics, thereby impeding chloride ion migration. Some inhibitor components may also interact or complex with chloride ions, reducing the concentration of free chloride in the pore solution. However, when the corrosion inhibitor dosage is increased to 3 wt% and 4 wt%, the retardation of cement hydration becomes more pronounced, resulting in a less compact pore structure and a corresponding increase in electric flux.

### 3.4. Water Absorption

**Figure 4:**
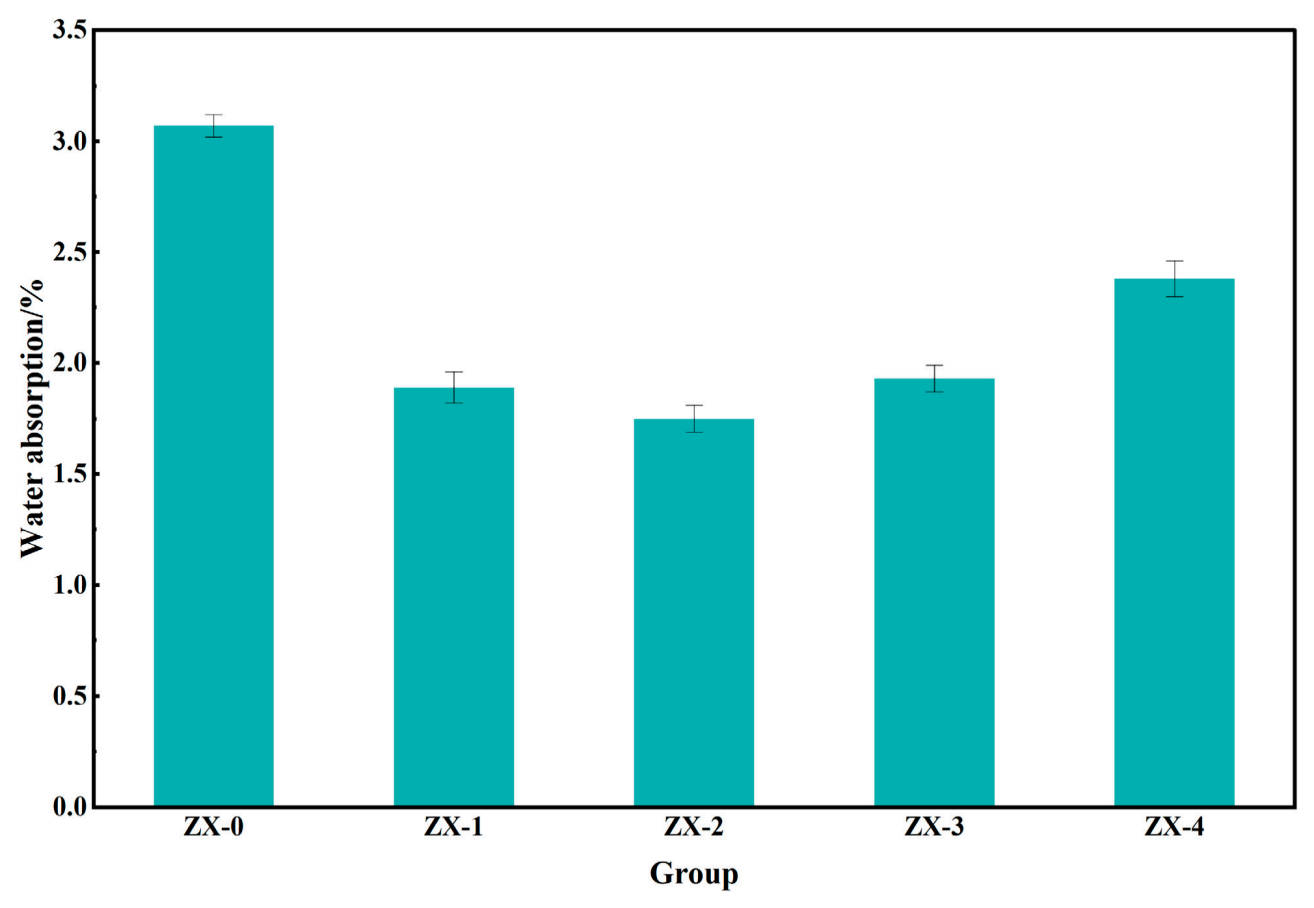
Effect of corrosion inhibitor dosage on water absorption of SCC.

The water absorption index is commonly used to evaluate the capillary water absorption behavior of unsaturated concrete and can directly reflect the compactness of the internal pore structure. Figure 4 shows the 28-day water absorption results of SCC specimens containing different dosages of corrosion inhibitor. One-way ANOVA indicates that the corrosion inhibitor dosage has a statistically significant effect on water absorption (*F*(4, 10) = 207.4, *p* < 0.001). Among all mixtures, ZX-2 exhibits the lowest water absorption of 1.75%, which is significantly lower than those of the other mixtures (*p* < 0.05). In comparison, the water absorption of the control mixture, ZX-0, is 3.07%. The incorporation of the corrosion inhibitor reduces the water absorption of all mixtures to varying degrees, indicating that it generally enhances the resistance of SCC to capillary water ingress. These results suggest that an appropriate dosage of corrosion inhibitor could promote a more uniform distribution of hydration products, facilitate the filling of capillary pores, and reduce connected porosity, thereby limiting the pathways available for capillary water transport. In addition, the polar functional groups in amino alcohol molecules may adsorb onto the pore surfaces, modify the wettability of the pore walls, and increase resistance to capillary water migration, further suppressing water absorption. However, at excessive dosages, the retarding effect of the corrosion inhibitor on cement hydration becomes more pronounced. Consequently, some capillary pores may not be filled promptly by hydration products, leading to an increase in water absorption.

### 3.5. Microstructure

#### 3.5.1. Low-Field Nuclear Magnetic Resonance Analysis

**Figure 5:**
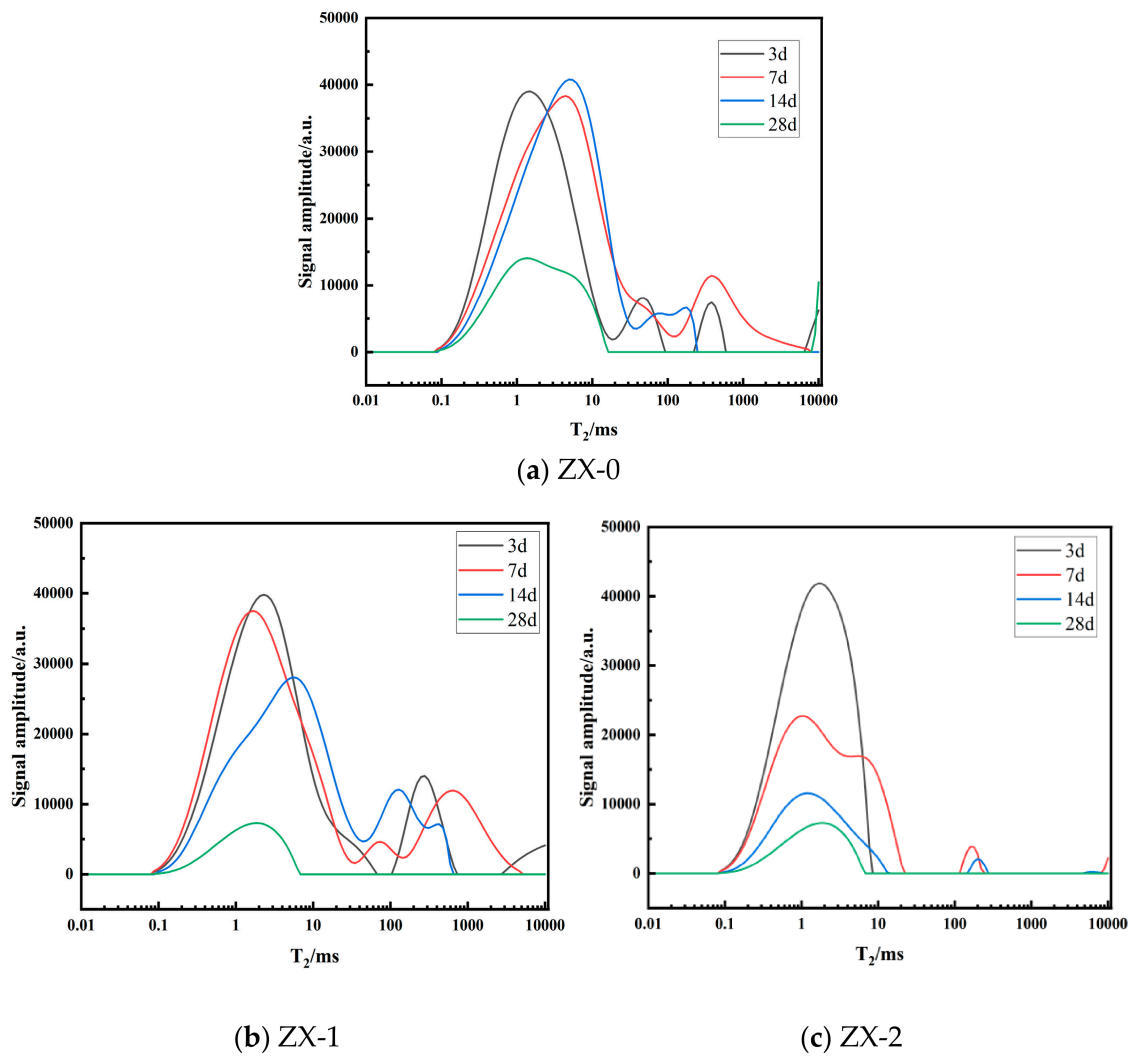

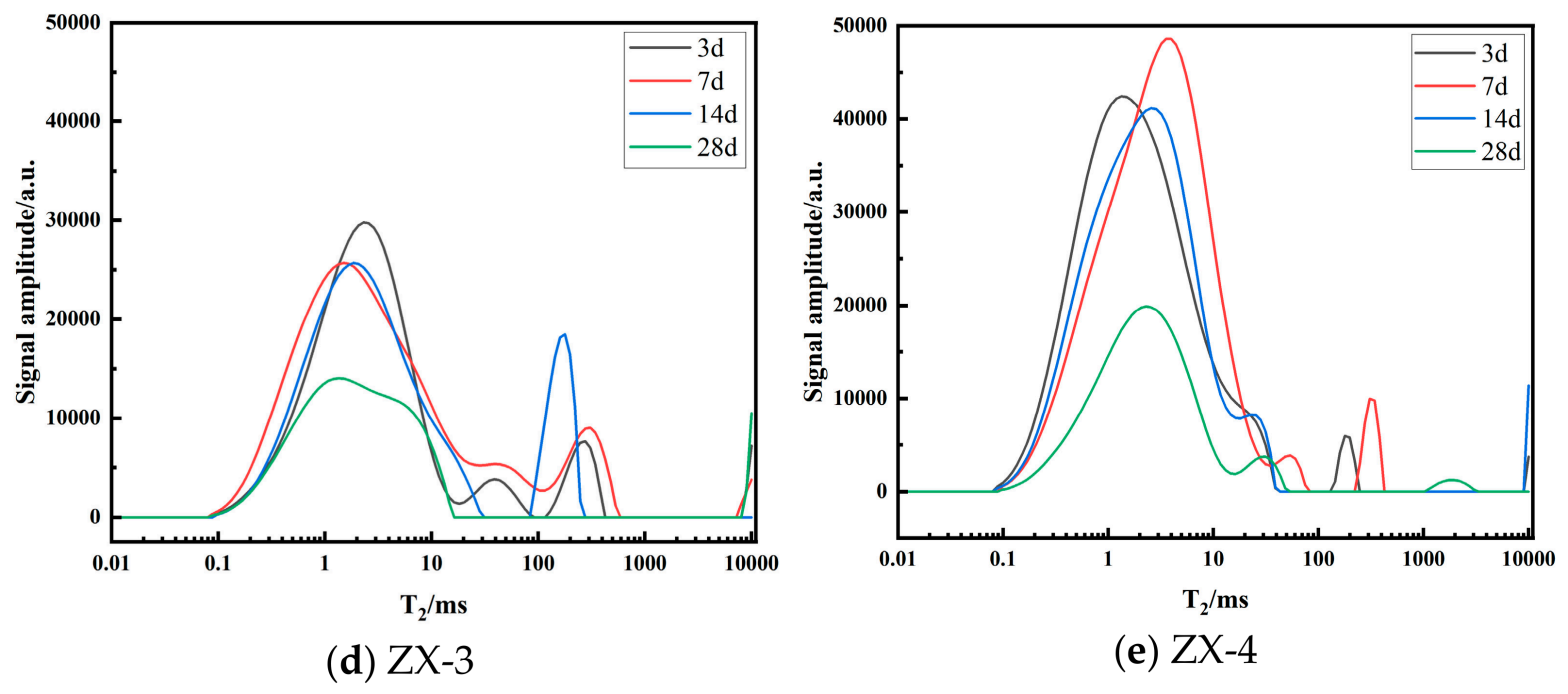
T_2_ relaxation-time spectra of SCC specimens with different corrosion inhibitor dosages after curing for 3, 7, 14, and 28 days.

Figure 5 presents the T_2_ relaxation-time spectra of SCC specimens containing different dosages of corrosion inhibitor at various curing ages. A shorter T_2_ relaxation time generally corresponds to a smaller pore size, whereas a longer relaxation time indicates larger pores. Accordingly, three distinct characteristic peaks can be identified in the T_2_ spectra, corresponding to gel pores, capillary pores, and macropores or macroscopic defects, respectively. At a curing age of 3 days, the T_2_ spectrum of ZX-0 exhibits pronounced peaks within the range of 100–1000 ms, indicating the presence of a considerable number of macropores or entrapped air voids at the early stage (Figure 5a). With increasing curing age, the spectral peaks progressively shift toward shorter relaxation times. The main peak gradually becomes concentrated within the range of 1–10 ms, while the peaks beyond 100 ms continuously decrease in intensity. This evolution indicates that ongoing cement hydration and the accumulation of hydration products progressively fill the internal pores, resulting in the refinement or closure of macropores.

As shown in Figure 5b, ZX-1 exhibits a slightly larger peak area above 100 ms at an early curing age than ZX-0, suggesting that the incorporation of the corrosion inhibitor slightly increases the proportion of early-age macropores. This behavior may be attributed to the adsorption of inhibitor molecules onto the surfaces of cement particles, which delays the early hydration process. However, as curing proceeds, the spectral peaks shift markedly toward shorter relaxation times. At 28 days, the pore structure is substantially refined, indicating that the adverse effect of the corrosion inhibitor on early hydration is partially offset by the continued formation and accumulation of hydration products.

For ZX-2, the T_2_ spectra exhibit a bimodal distribution at 7 d and 14 d, followed by a return to a unimodal distribution at 28 d, accompanied by a reduction in signal intensity (Figure 5c). This evolution suggests that the pore structure is initially refined, undergoes slight coarsening at the intermediate stage, and is subsequently densified at the later stage. Meanwhile, the decrease in overall signal intensity indicates a reduction in total porosity. These observations suggest that an appropriate dosage of corrosion inhibitor can effectively limit the formation of early-age macropores and promote subsequent pore refinement. At 28 d, the T_2_ spectrum displays a typical unimodal distribution, with the main peak located entirely within the range of 0.1–10 ms. This indicates that the pore system is dominated by fine gel pores, while the proportions of capillary pores and macropores are substantially reduced. Therefore, the incorporation of 2 wt% corrosion inhibitor promotes a more uniform distribution of hydration products and optimizes the pore structure of SCC.

With a further increase in corrosion inhibitor dosage, the peak area above 100 ms increases markedly at early curing ages, as shown in Figure 5d,e. This result indicates that an excessive dosage of corrosion inhibitor strongly suppresses early cement hydration, thereby increasing the initial porosity and promoting the formation of macropores. Although continued hydration may subsequently refine the pore structure, the pronounced early-age retardation limits the overall densification of the matrix.

#### 3.5.2. X-CT Analysis

**Figure 6:**
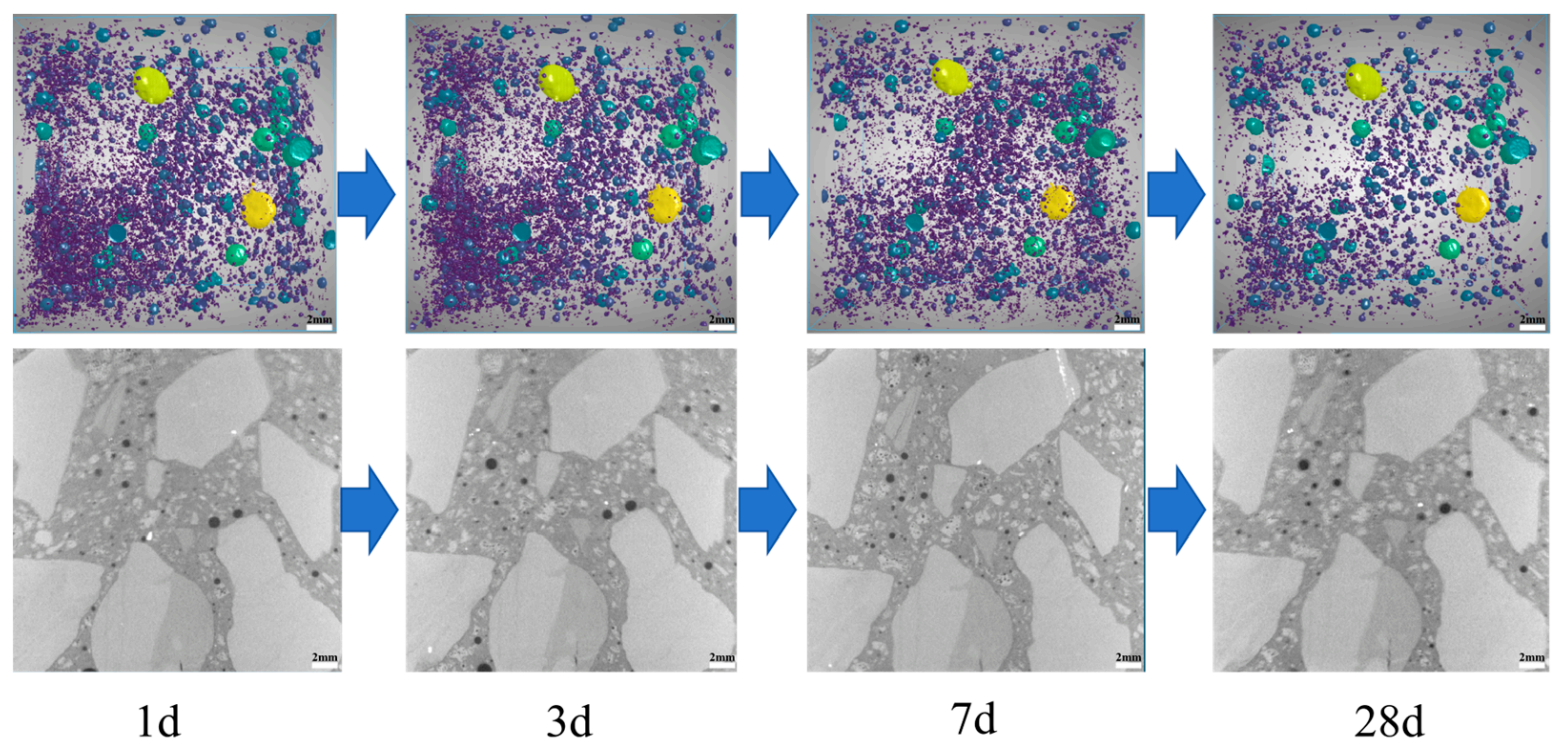
X-CT images of SCC specimens with a corrosion inhibitor dosage of 0 wt% at 1, 3, 7, and 28 days.

**Figure 7:**
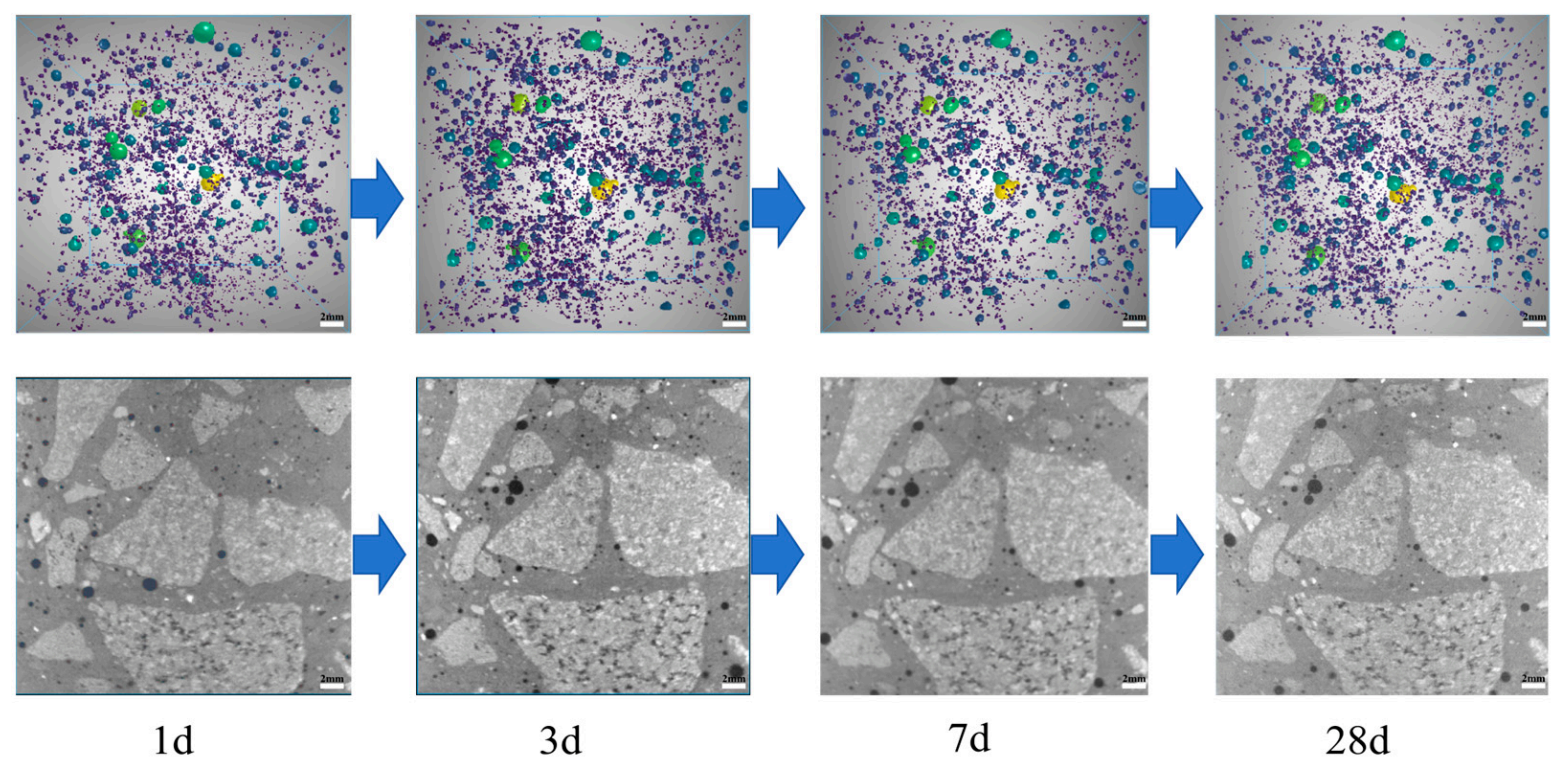
X-CT images of SCC specimens with a corrosion inhibitor dosage of 1 wt% at 1, 3, 7, and 28 days.

**Figure 8:**
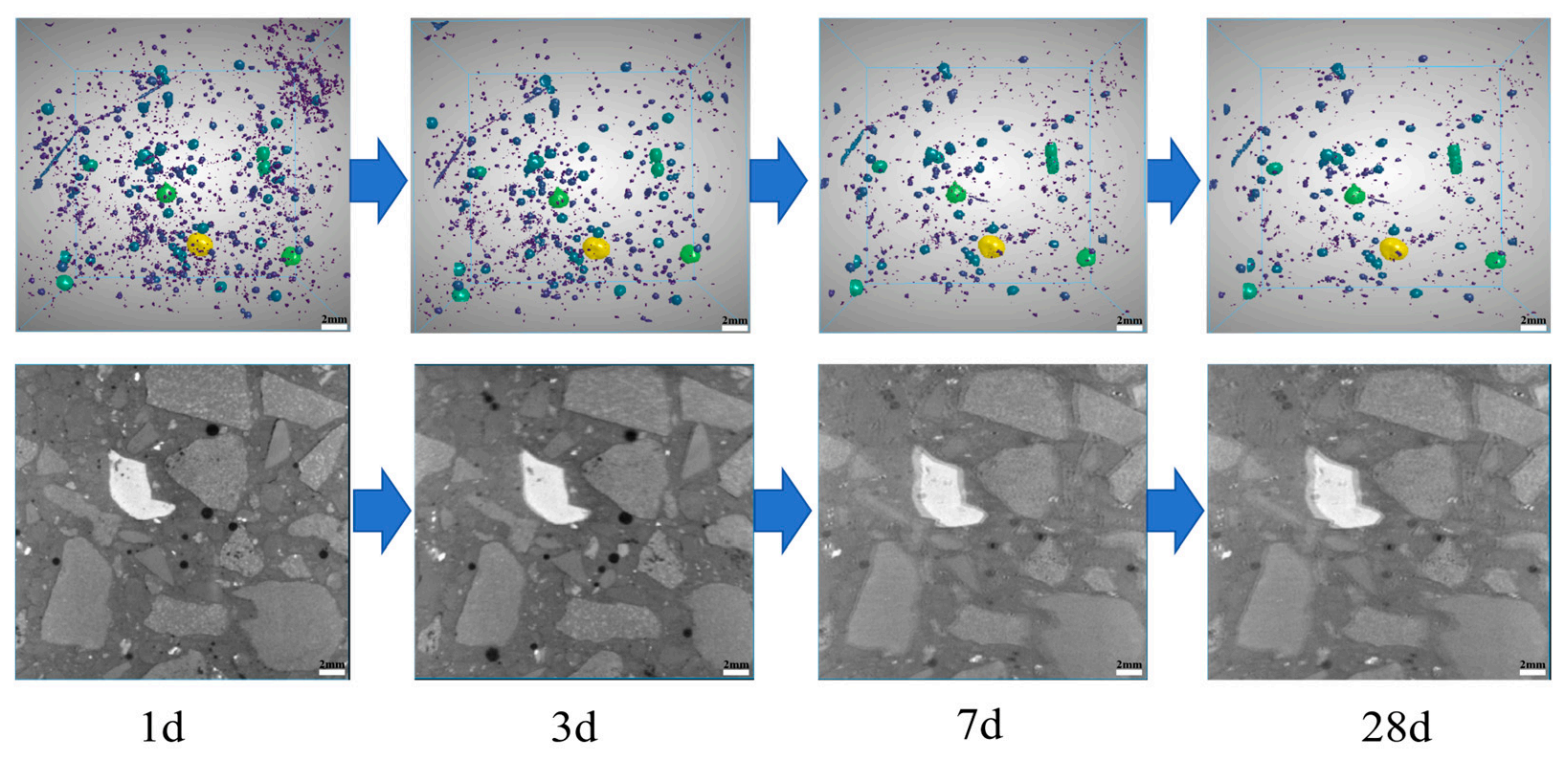
X-CT images of SCC specimens with a corrosion inhibitor dosage of 2 wt% at 1, 3, 7, and 28 days.

**Figure 9:**
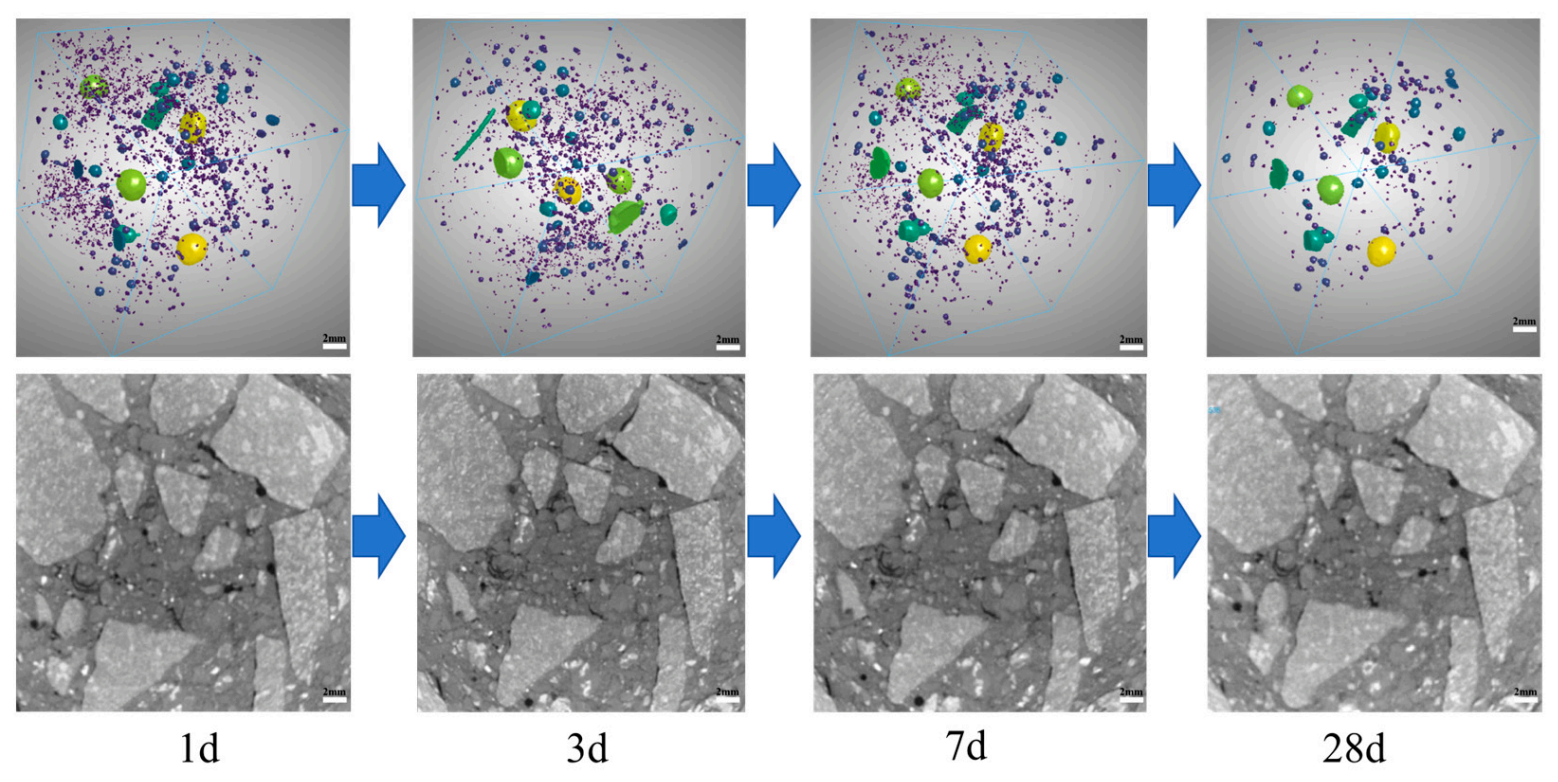
X-CT images of SCC specimens with a corrosion inhibitor dosage of 3 wt% at 1, 3, 7, and 28 days.

**Figure 10:**
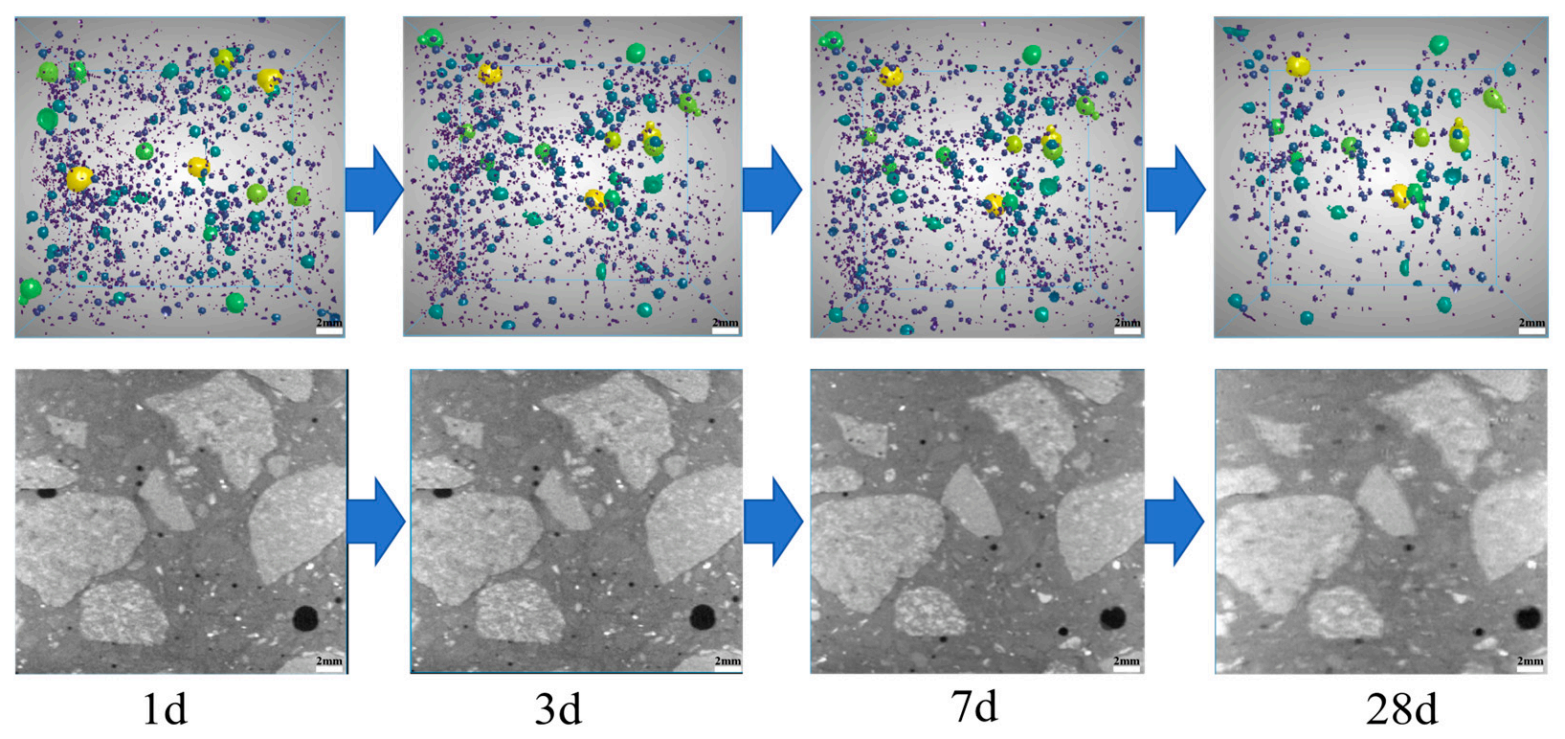
X-CT images of SCC specimens with a corrosion inhibitor dosage of 4 wt% at 1, 3, 7, and 28 days.

X-CT was employed to reconstruct and analyze the internal pore structure of SCC specimens (30 mm × 30 mm × 30 mm) containing different dosages of corrosion inhibitor after curing for 1, 3, 7, and 28 days. Figure 6, Figure 7, Figure 8, Figure 9 and Figure 10 show the three-dimensional pore distribution and representative two-dimensional cross-sectional images of the specimens at different curing ages. The two-dimensional images reveal no substantial differences in the overall morphology among specimens with different corrosion inhibitor dosages or curing ages. Moreover, no evident macrocracks or interconnected defects are observed. These findings indicate that all specimens exhibit satisfactory casting quality and that the incorporation of the corrosion inhibitor does not cause visible structural damage.

The three-dimensional images show that the number of pores in all groups gradually decreases with increasing curing age, reflecting the progressive filling and refinement of the internal pore network by cement hydration products. In addition, the corrosion inhibitor dosage has a pronounced influence on both pore size distribution and pore quantity. Notably, a small number of large pores are observed in the ZX-3 specimens. Although these pores are limited in number, their equivalent diameters are substantially greater than those observed in the other groups. In contrast, ZX-2 exhibits smaller macropores and a markedly lower number of large pores than ZX-3. The pore distribution in ZX-2 is also more uniform and refined.

These results indicate that the incorporation of 2 wt% corrosion inhibitor effectively suppresses the formation of large pores while promoting the filling and refinement of small pores and capillary pores by hydration products. Although the addition of 3 wt% corrosion inhibitor still contributes to a reduction in total porosity, its adverse effect on cement hydration becomes more pronounced. The resulting localized delay or insufficiency in hydration promotes the formation of a limited number of isolated macropores with relatively large equivalent diameters.

**Figure 11:**
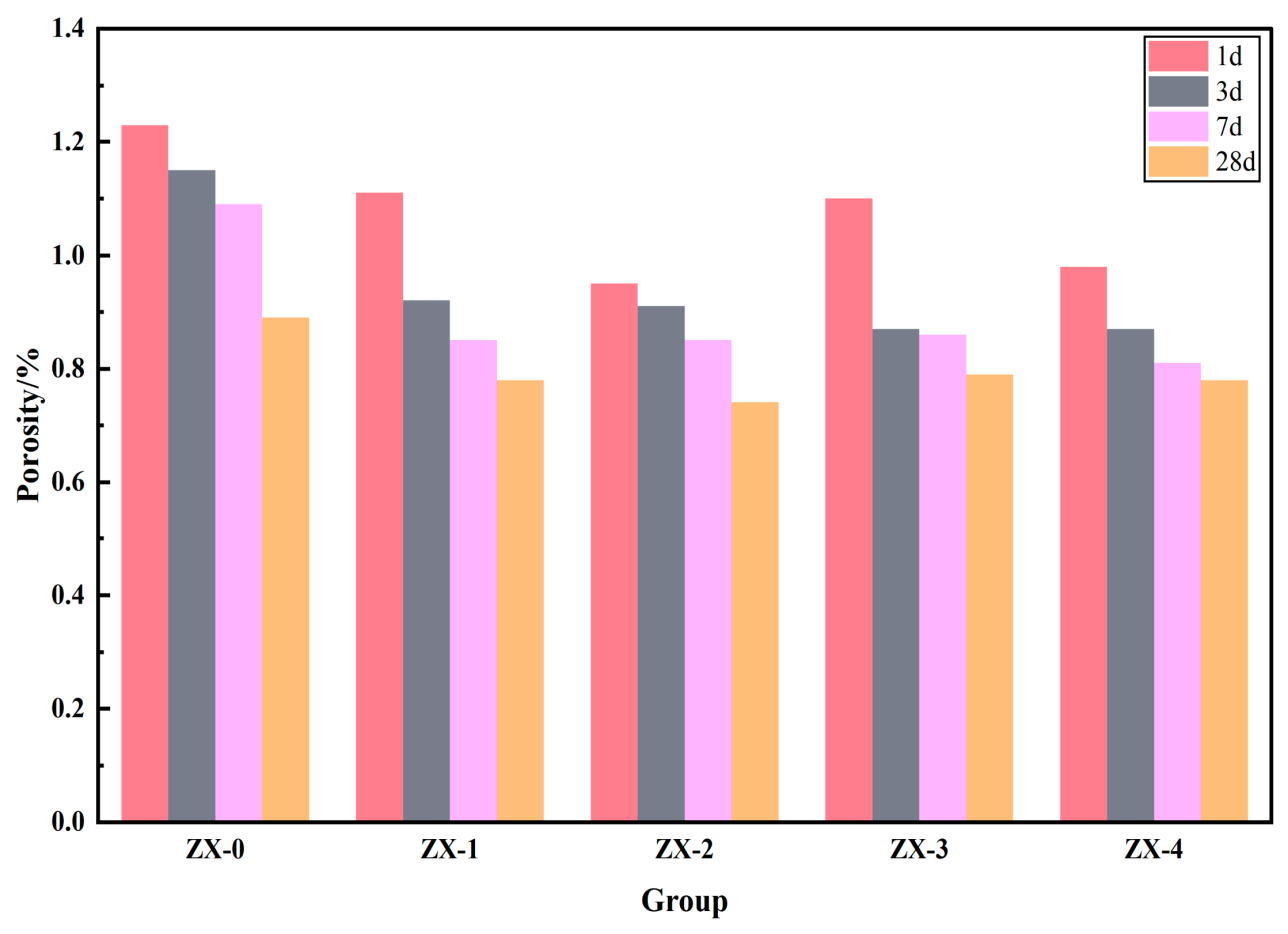
Total porosity of SCC specimens with different corrosion inhibitor dosages at different curing ages.

Figure 11 summarizes the effects of corrosion inhibitor dosage on the total porosity of SCC under standard curing conditions. All mixtures containing the corrosion inhibitor exhibit lower porosity than the control mixture, ZX-0, indicating that the incorporation of the corrosion inhibitor generally contributes to the optimization of the pore structure of SCC. The total porosity increases in the following order: ZX-2 < ZX-1 < ZX-4 < ZX-3 < ZX-0. Among all the mixtures, ZX-2 exhibits the lowest porosity and the most pronounced reduction in macropore content, demonstrating the strongest pore-refinement effect. Although the porosities of ZX-3 and ZX-4 are higher than that of ZX-1, they remain substantially lower than that of ZX-0.

These results indicate that an appropriate dosage of corrosion inhibitor most effectively optimizes the pore structure. Although an excessive dosage can still reduce the total porosity relative to the control mixture, its pore-refinement effect becomes less pronounced. This behavior may be primarily attributed to the amino alcohol components of the corrosion inhibitor, which adsorb onto the surfaces of cement particles, regulate the spatial distribution of hydration products, and facilitate the uniform filling of pores by C-S-H gel. However, an excessive dosage of corrosion inhibitor markedly retards early cement hydration and reduces the rate of hydration-product formation. Consequently, some large pores cannot be filled promptly, thereby weakening the overall pore-refinement effect.

**Figure 12:**
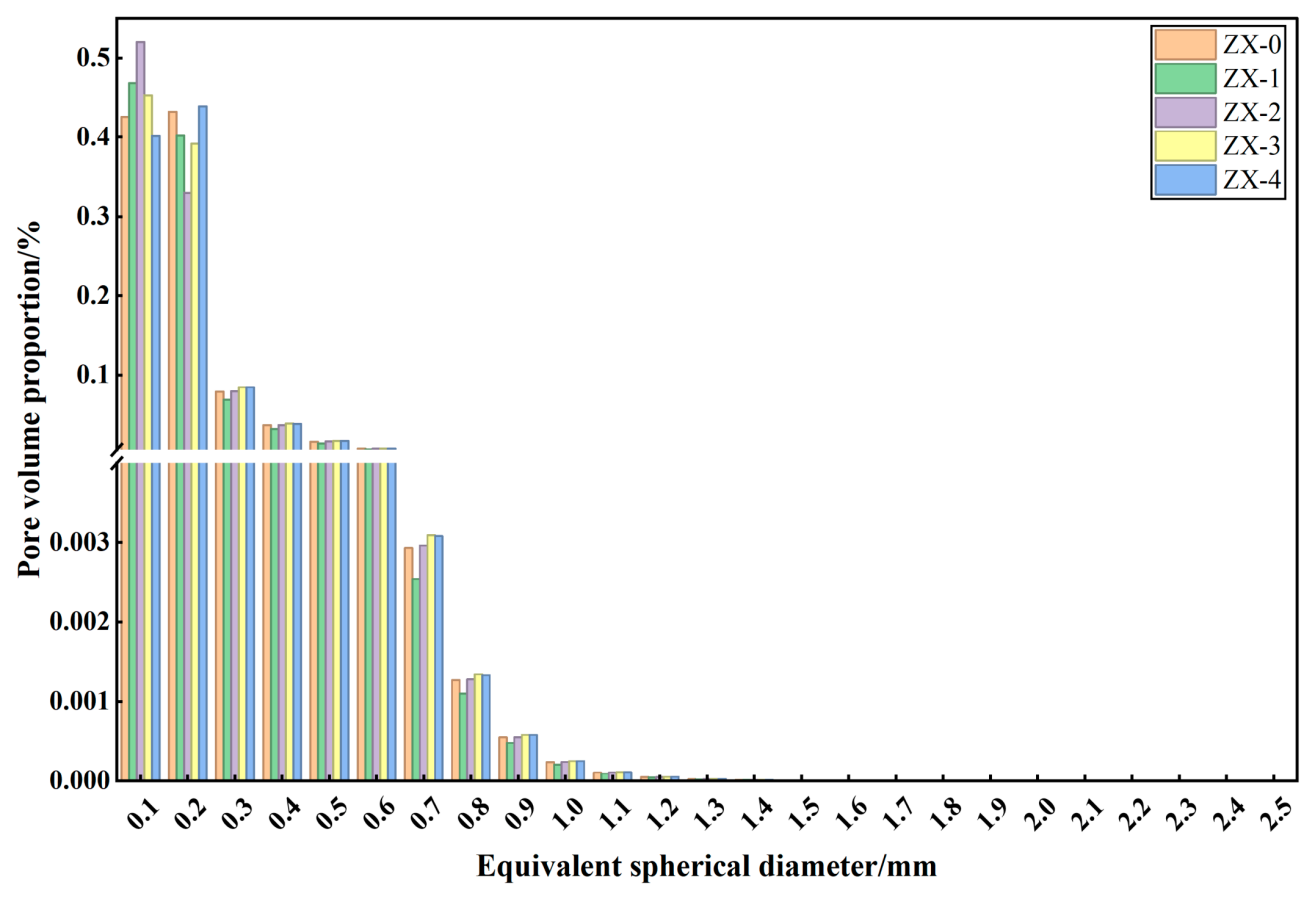
Pore volume proportions within different equivalent spherical diameter ranges of SCC specimens with different corrosion inhibitor dosages after 28 days of curing.

Figure 12 presents the proportions of pores within different equivalent spherical diameter ranges for SCC specimens containing different corrosion inhibitor dosages after 28 days of curing. For all specimens, pores with an equivalent spherical diameter of 0–0.2 mm constitute the dominant fraction, accounting for approximately 84–87% of the total pore volume. Among the mixtures, ZX-2 exhibits the highest proportion of pores in the 0–0.1 mm range, reaching 52.01%, whereas the proportion of pores in the 0.1–0.2 mm range is only 32.99%. This distribution indicates that the incorporation of 2 wt% corrosion inhibitor produces the most pronounced pore-refinement effect. In contrast, ZX-4 exhibits the lowest proportion of pores in the 0–0.1 mm range, at 40.12%, while the proportion of pores in the 0.1–0.2 mm range reaches 43.89%. The shift in pore proportion from the smaller-diameter range to the larger-diameter range indicates an evident coarsening of the pore structure in ZX-4.

Based on the total porosity obtained from the X-CT reconstruction, correlation analyses were conducted between total porosity and the 28-day compressive strength, electric flux, and water absorption. Total porosity is positively correlated with electric flux and water absorption but negatively correlated with compressive strength. The corresponding coefficients of determination (R^2^) are 0.89, 0.93, and 0.91, respectively. These strong correlations quantitatively demonstrate that pore refinement plays a critical role in improving the mechanical properties and durability of SCC. The denser microstructure of ZX-2, characterized by the lowest total porosity and the highest proportion of small pores within the 0–0.1 mm range, directly contributes to its superior mechanical and durability performance.

#### 3.5.3. SEM Observations

**Figure 13:**
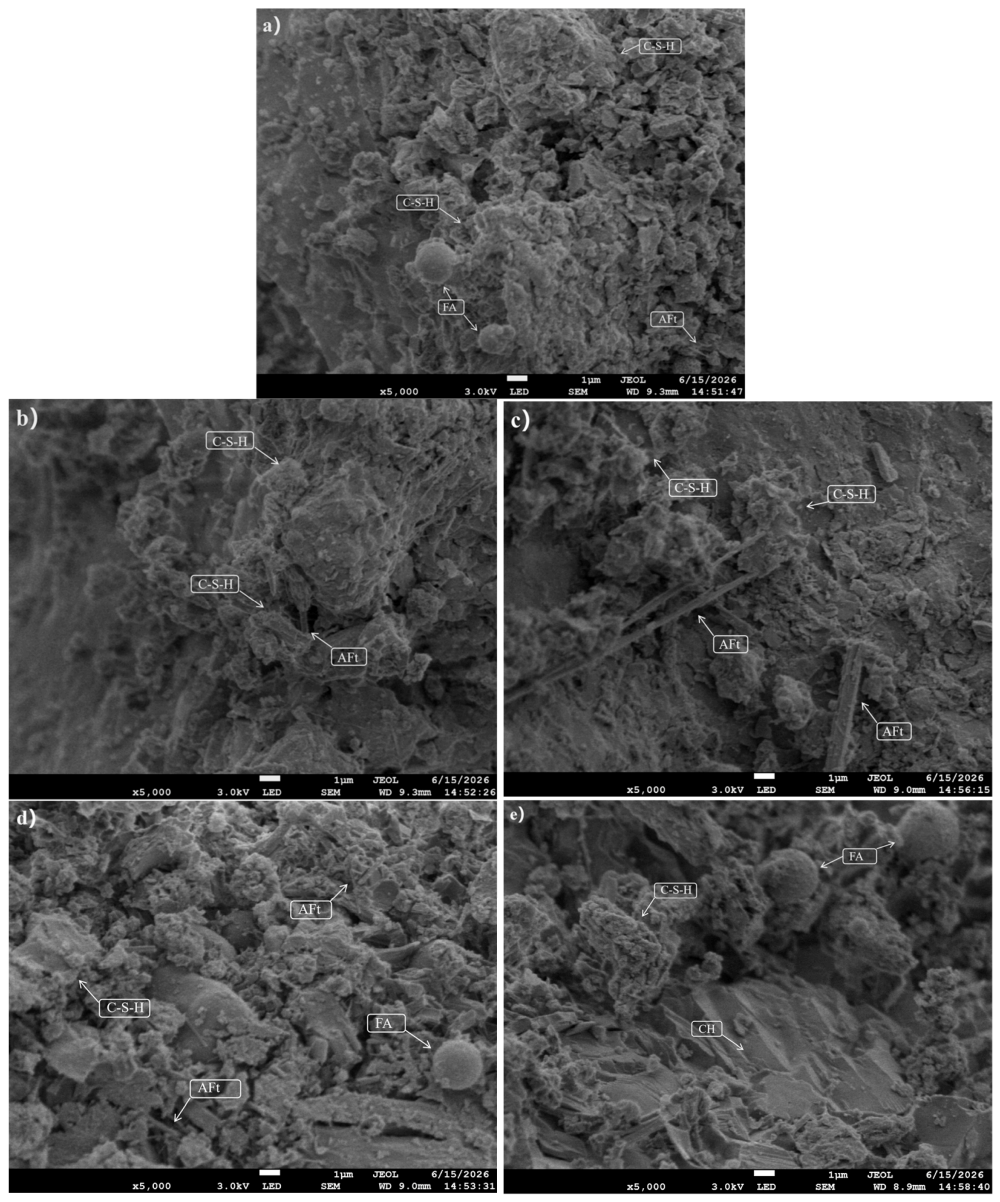
SEM images of SCC specimens with different corrosion inhibitor dosages after 28 days of curing. (**a**) ZX-0; (**b**) ZX-1; (**c**) ZX-2; (**d**) ZX-3; (**e**) ZX-4.

To further elucidate the effect of corrosion inhibitor dosage on the microstructure of SCC, SEM observations were conducted on SCC specimens after 28 days of curing, as shown in Figure 13a–e. In the ZX-0 specimen, flocculent C-S-H gel and a small amount of needle-like ettringite (AFt) are observed. However, loosely structured porous regions remain within the matrix, and the hydration products are distributed relatively unevenly. After the incorporation of 1 wt% corrosion inhibitor, the proportion of C-S-H gel increases, and the matrix exhibits an initial tendency toward densification. This result indicates that a low dosage of corrosion inhibitor does not markedly disrupt the cement hydration system and may improve the spatial distribution of hydration products. When the corrosion inhibitor dosage is increased to 2 wt%, the specimen develops a comparatively dense and homogeneous microstructure, with hydration products more uniformly distributed throughout the matrix. However, when the corrosion inhibitor dosage is further increased to 3 wt% or higher, the uniformity of the hydration products decreases significantly. In addition to abundant C-S-H gel, a large number of calcium hydroxide (CH) crystals and partially unreacted fly ash (FA) particles are observed in the matrix.

### 3.6. Mechanism Analysis

The primary active components of the corrosion inhibitor are triethanolamine and triisopropanolamine, together with small amounts of phosphate-based compounds. These organic amines possess surface-active properties and can adsorb onto the surfaces of cement particles, thereby slightly retarding the early hydration of C_3_S and C_2_S [23]. At the same time, the adsorption of amino alcohol molecules alters the surface charge and interfacial tension of cement particles, promoting more uniform dispersion of cement particles. This dispersing effect may facilitate a more homogeneous spatial distribution of hydration products and reduce localized accumulation or deficiency.

Consequently, although an appropriate dosage of corrosion inhibitor may slightly delay early hydration and lead to a modest reduction in strength, the more uniformly distributed hydration products may fill internal pores more effectively and improve the compactness of the matrix. However, when the corrosion inhibitor dosage is increased to 3 wt% or 4 wt%, excessive adsorption of amino alcohol molecules on the surfaces of cement particles may intensify the retardation of hydration. As a result, the formation rate of hydration products decreases, preventing some pores from being filled in a timely manner and leading to the development of locally loose and porous regions.

## 4. Conclusions

This study systematically investigates the effects of corrosion inhibitor dosage on the fresh properties, mechanical properties, durability, and pore structure of SCC, as well as the associated mechanisms and microstructural evolution. The main conclusions are as follows.

At a corrosion inhibitor dosage of 2 wt%, the adverse effects of the corrosion inhibitor on the mechanical properties of SCC are minimized while satisfactory workability is maintained. The compressive and splitting tensile strengths of ZX-2 at different curing ages remain the highest among the corrosion-inhibitor-containing mixtures.

An appropriate corrosion inhibitor dosage also improves the compactness of the concrete matrix, substantially reduces the electric flux and capillary water absorption, and enhances the resistance of SCC to chloride ion penetration and capillary water ingress. The combined LF-NMR, X-CT, and SEM results indicate that specimens containing 2 wt% of corrosion inhibitor exhibit the lowest total porosity and the highest proportion of fine pores. The inhibitor may effectively refine the pore structure, reduce the total porosity, suppress the formation of porous defects, and promote the development of a relatively dense microstructure.

Overall, a corrosion inhibitor dosage of 2 wt% provides the most favorable balance among workability, mechanical properties, durability, and microstructural compactness under the conditions investigated. These findings provide experimental evidence and technical guidance for the safe and effective application of corrosion inhibitors in SCC structures.

Nevertheless, this study has several limitations. Hydration heat evolution and phase composition were not characterized; therefore, the hydration-regulation mechanism of the corrosion inhibitor could not be quantitatively established. In addition, the effects of the corrosion inhibitor on the long-term durability of SCC and the long-term corrosion protection of embedded steel reinforcement were not systematically evaluated. Future studies should combine isothermal calorimetry, quantitative phase analysis, and long-term exposure testing to clarify the hydration mechanism and assess the durability and corrosion-protection performance over extended service periods.

## Figures and Tables

**Table 1:** Mix proportions for SCC (kg/m^3^).

Group	Cement	Fly Ash	Slag	Sand	Gravel	Water	Water Reducer	Corrosion Inhibitor
ZX-0	264	120	96	720	1080	158	4.8	0
ZX-1	261.36	118.8	95.04	720	1080	158	4.8	4.8
ZX-2	258.72	117.6	94.08	720	1080	158	4.8	9.6
ZX-3	256.08	116.4	93.12	720	1080	158	4.8	14.4
ZX-4	253.44	115.2	92.16	720	1080	158	4.8	19.2

## Data Availability

The original contributions presented in this study are included in the article. Further inquiries can be directed to the corresponding authors.
